# Creeping Fat in Crohn’s Disease—Surgical, Histological, and Radiological Approaches

**DOI:** 10.3390/jpm13071029

**Published:** 2023-06-21

**Authors:** Ioanna Aggeletopoulou, Efthymios P. Tsounis, Athanasia Mouzaki, Christos Triantos

**Affiliations:** 1 Division of Gastroenterology, Department of Internal Medicine, University Hospital of Patras, 26504 Patras, Greece; iaggel@hotmail.com (I.A.); makotsouno@gmail.com (E.P.T.); 2 Division of Hematology, Department of Internal Medicine, Medical School, University of Patras, 26504 Patras, Greece; mouzaki@upatras.gr

**Keywords:** Crohn’s disease, creeping fat, fat wrapping, adipose tissue, mesenteric adipose tissue, histological characteristics, imaging data, artificial intelligence

## Abstract

During the course of Crohn’s disease, the response of mesenteric adipose tissue to the production of inflammatory mediators and bacterial invasion through the intestinal mucosa results in the formation of creeping fat. Creeping fat describes the arresting finger-like projections that surround the inflamed bowel. In this review, the microscopic and macroscopic features of creeping fat and histological evidence for the importance of this tissue are discussed. Moreover, the most recent insights into the radiological assessment of creeping fat in patients with Crohn’s disease are reported. Advances in imaging techniques have revolutionized the possibility of visualization and quantification of adipose tissue depots with excellent accuracy. Visceral fat has been significantly correlated with various Crohn’s-disease-related outcomes. Despite the difficulties in distinguishing physiologic perienteric fat from creeping fat, the growing interest in fat-wrapping in Crohn’s disease has rejuvenated radiologic research. With regard to the noninvasive fat-wrapping assessment, a novel CT enterography-based mesenteric creeping fat index has been developed for the mitigation of the confounding effect of normal retroperitoneal and perienteric adipose tissue. Research on machine learning algorithms and computational radiomics in conjunction with mechanistic studies may be the key for the elucidation of the complex role of creeping fat in Crohn’s disease.

## 1. Introduction

Crohn’s disease is a chronic inflammatory bowel disease that can affect any part of the gastrointestinal tract and is characterized by periods of flare-ups and remission [1]. It is considered a heterogenous multifactorial disease in which genetic environmental, immunological, and microbial factors contribute to disease pathogenesis. In parallel, there is ongoing research into potential gender differences and biases related to certain aspects of the disease [2,3,4,5,6,7]. The management of patients with Crohn’s disease depends on the severity of the disease, the patient’s risk assessment, the patient’s preferences, and clinical parameters [8]. Treatment options encompass a range of interventions, including the use of steroids, monoclonal antibody therapies, immunomodulators, and surgical procedures [8]. Additionally, proper nutritional management affects the overall treatment and well-being of individuals with Crohn’s disease [9,10,11,12,13,14].

The phenomenon of hypertrophied adipose tissue surrounding inflamed intestinal segments in Crohn’s disease was first described by Burril Crohn and colleagues in 1932 [15]. Almost a century later, the function of this pathologic entity, called “creeping fat” or “fat-wrapping”, and the mechanisms mediating its formation are not yet fully deciphered. Emerging evidence has presented the multiple functions of adipose tissue beyond energy storage, bringing creeping fat to the forefront of scientific research. Adipose tissue constitutes a complex and highly active endocrine and metabolic organ which critically contributes to the regulation of immunity and interferes with inflammatory signaling cascades [16]. Adipose tissue is composed of a wide variety of cell types including adipocytes, immune cells, endothelial cells, pre-adipocytes, fibroblasts, and stem cells [17]. Even though adipocytes are responsible for the great majority of the fat pad volume (>90%), they constitute only about 20–40% of the cellular content [18].

**Figure 1:**
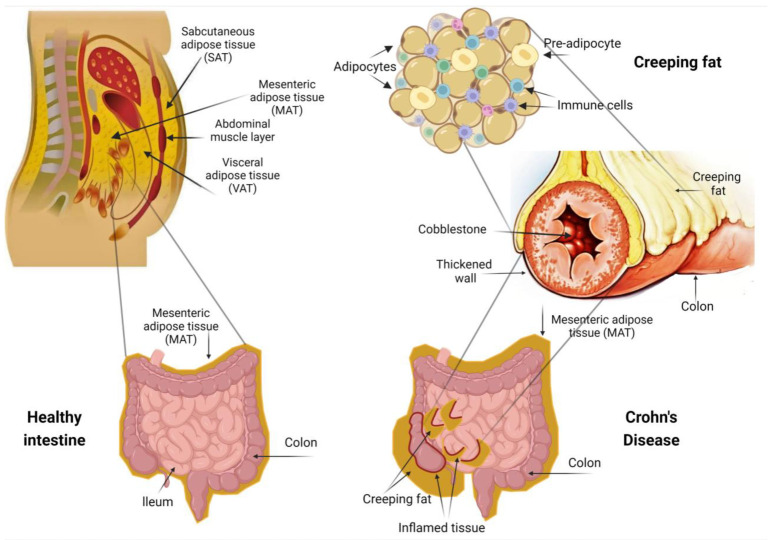
Creeping fat, a hallmark of Crohn’s disease, consists of adipose tissue located around the inflamed intestinal segments. The complex interaction between adipose tissue and immune cells during inflammatory responses remains elusive. Creeping fat is composed of small hyperplastic adipocytes, adipocyte progenitors, and various immune cells such as macrophages, T cells, and B cells. These cells produce pro- and anti-inflammatory mediators such as cytokines, fatty acids, and growth factors, which contribute to the mesenteric fat function, thus modulating the immune responses and the intestinal inflammation. This figure was generated using BioRender, available online at: https://biorender.com (accessed on 2 February 2023).

In Crohn’s disease, dysbiosis and transmural injury compromise the integrity of the intestinal barrier, resulting in an excessive influx of intraluminal microbiota and xenobiotics [19]. The gut and the peri intestinal fat present a close anatomic relationship, which implies a direct reciprocal immunologic association, whereas adipocytes are equipped with a great number of innate immunity sensors that respond to invading stimuli [20]. As a result, adipocytes and their progenitor cells undergo major immunophenotypic alterations, leading to adipose tissue remodeling and the formation of creeping fat (Figure 1) [21].

Although, creeping fat was historically considered an innocent bystander, it is actually an active player during inflammation and immunity [22]. Creeping fat is an immunologically active organ that produces various pro- and anti-inflammatory cytokines, pro-fibrotic factors, and adipokines, serving as a regulator of paracrine/autocrine signaling and a modulator of immune responses [23]. In parallel, the creeping-fat-derived adipocytes in Crohn’s disease produce higher levels of total, saturated, and polyunsaturated free fatty acids compared with the mesenteric fat in ulcerative colitis and healthy individuals, substantially increasing the intestinal smooth muscle cell proliferation [24].

The contribution of creeping fat to inflammatory signaling partially explains the reason of being related to more progressive Crohn’s disease or a more intricate disease phenotype [25,26,27]. Ha et al. [28] disclosed that in Crohn’s disease the increased mucosal-related gut bacteria translocate into the mesenteric adipose tissue, resulting in the expansion of this fat tissue, suggesting that creeping fat may restrict systemic bacterial spreading. In parallel, data suggest an alternative immunoregulatory role of creeping fat as a second barrier that impedes the development of a systemic inflammatory response at the expense of a progressively proliferating pro-fibrotic environment [24,29,30]. Additional insight to the previously published single-cell RNA sequencing data derived by Ha et al. was provided by Shu et al. [31], which described the cellular heterogeneity in the mesenteric adipose tissue of patients with Crohn’s disease. In creeping fat, a specific stromal vascular cell fraction has been found, exhibiting high expression of the lipoprotein lipase [31]. This subpopulation displays high transcriptional activity of peroxisome proliferator-activated receptor γ (PPARγ) and is closely involved in the upregulation of the PPAR signaling pathway in the metabolism of lipids and in antibacterial responses [31]. One more abundant subpopulation which was also described in this study is the fibroblast subpopulation (FC3); this cell subset is closely related to inflammatory responses and intestinal fibrosis. Lasty, various macrophage subclusters within the myeloid compartment have been also reported [31].

In the current review, the microscopic and macroscopic features of creeping fat and histological evidence for the importance of this tissue are described in detail. In parallel, emphasis is placed on the most recent insights into the radiological assessment of creeping fat in patients with Crohn’s disease.

## 2. Anatomical Relationship between Mesenteric Adipose Tissue and the Intestinal Wall

The mesentery is formed by a double fold of the peritoneum and attaches the gastrointestinal tract to the posterior abdominal wall. It provides topographic stability and some flexibility necessary for normal gut motility [32]. The major histologic components of the mesentery include the surface mesothelium, which is supported by a thin layer of loose, fibrous connective tissue overgrown by adipocytes [33]. Conventional speculation about the fragmented nature of the mesentery is now considered outdated, as recent advances have demonstrated that the mesentery is a unique sheet-like structure that extends in continuity from the duodenojejunal flexure to the rectum [32,34]. The mesentery and the intestine have a close anatomical relationship that is established in the early stages of embryogenesis. While the epithelial component of the gut originates from the endoderm, the mesenchymal cells originate from the mesentery [24,32,33]. The mesothelium of the mesentery merges into the serosa and contributes to the cell population of this layer [34]. In parallel, bundles of connective tissue extend from the mesentery into the underlying outer layers of the intestinal wall, including the muscularis mucosa and submucosa, forming a continuity at their intersection [32,33]. Lymphatic and blood vessels crossing this border allow unimpeded transport of immune cells and signaling molecules from the mesentery to the gut and vice versa. This partly explains the propensity for polarized ulceration, which in Crohn’s disease primarily affects the mesenteric rather than the antimesenteric border of the intestine [35]. Conversely, the complicated cross-talk mediated by neuropeptides, adipokines, and vascular/lymphatic endothelia may facilitate adipose tissue remodeling near the inflamed bowel wall [36]. Indeed, the impaired barrier function of mesenteric lymphatics in Crohn’s disease, due to architectural disorganization and tight junction impairment, results in an influx of proinflammatory mediators and other immunostimulatory lymphatic components that may promote the formation of creeping fat [37]. Clearly, the true histologic overlap at the hilum, which extends throughout the intestine from the duodenum to the rectum, is clinically relevant in Crohn’s disease and provides the basis for an interaction between mesenchymal adipose tissue and intestinal inflammation.

## 3. Role of Mesenchymal Stem Cells in the Adipose Tissue

Mesenchymal stem cells (MSCs), a type of multipotent cells, can be found in various tissues, including adipose tissue [38,39]. The role of mesenchymal stem cells in fat tissue has been the subject of extensive research, as they have been implicated in various key aspects such as adipogenesis, fat mass regulation, immunomodulation, tissue repair, and regeneration [40,41,42]. In recent years, there has been increasing interest in the potential therapeutic role of MSCs in the management of Crohn’s disease [43,44]. MSCs have the ability to differentiate into adipocytes, the cells responsible for storing fat in the body. Adipogenesis refers to the process by which MSCs mature into adipocytes, contributing to the formation and growth of fat tissue [45]. MSCs in fat tissue contribute to the maintenance of the balance between fat storage and fat release [46]. They can respond to body signals, such as hormonal cues, to either promote or inhibit the accumulation of fat mass [46]. MSCs possess immunomodulatory properties, regulating immune responses; in fat tissue, MSCs modulate immune cell activity, promoting an anti-inflammatory environment [46]. This can impact the overall function and health of adipose tissue. Nevertheless, under pathological conditions, adipose-derived mesenchymal stem cells (ASCs) can demonstrate pro-inflammatory characteristics and attract inflammatory immune cells in their microenvironment [47]. As a result, an inflammatory microenvironment is induced, contributing to the dysfunction of ASCs. In the context of Crohn’s disease, ASCs derived from mesenteric or subcutaneous adipose tissue demonstrate distinct characteristics compared with healthy MSCs [21]. These Crohn’s disease-associated ASCs exhibit an inflammatory, proliferative, and invasive profile, along with impaired adipogenic capacities and immunomodulatory properties [21]. Additionally, Crohn’s-disease-related ASCs show greater ability in bacterial phagocytosis and migration, as well as increased expression of HLA class II molecules [21]. The invasive phenotype of ASCs in patients with Crohn’s disease is mediated by an inflammasome-induced inflammatory state; inhibition of inflammasome signaling reverses this characteristic [21]. These findings suggest that ASCs derived by patients with Crohn’s disease may play a role in inflammation-induced damage to intestinal tissues [21].

In parallel, MSCs have regenerative capabilities and contribute to tissue repair [42]. In fat tissue, MSCs take part in the renewal and regeneration of adipocytes, an important procedure in maintaining the functionality and integrity of fat depots [48]. In Crohn’s disease, MSCs in adipose tissue have the potential to differentiate into cells that can repair damaged intestinal tissue and promote healing of the inflamed gut [49]. The unique properties of MSCs, including their regenerative and immunomodulatory properties, make them promising candidates for various therapeutic applications. Researchers are exploring the use of MSCs from adipose tissue in regenerative medicine, tissue engineering, and the treatment of various conditions, including metabolic disorders and inflammatory diseases [47,49]. Specific mechanisms and functions of MSCs in fat tissue are still being investigated, and their full potential in therapeutic applications has yet to be realized. Regarding the position of MSC treatment options in the therapeutic armamentarium of Crohn’s disease, it seems promising; however, more studies are needed to better understand the optimal administration methods, dosage, and long-term effects.

## 4. The Role of Creeping Fat in Intestinal Inflammation

The creeping-fat-related structural disorganization involves inflammatory lesions, fibrotic features accompanied by abnormal collagen fiber deposition, and augmented necrosis of adipocytes [23]. Molecular profiling of creeping fat has demonstrated a profound increase in the expression of inflammation-associated genes, whereas genes associated with lipid metabolism have been found to be downregulated [50]. In a mouse model featuring creeping fat, an overexpression of inflammatory markers and a decrease in mature adipocytes, accompanied by a prevalence of preadipocytes and fibroblast-like cells, have been observed [51]. In parallel, fat depots in the mesentery have been found to contribute to Crohn’s-disease-associated inflammation, specifically through the Substance P (SP)- neurokinin receptor 1 (NK-R1) signaling pathway [52]. Preadipocytes derived from individuals with Crohn’s disease exhibited increased sensitivity to SP and displayed a distinct pattern of cytokine secretion compared with cells from healthy controls [52]. Notably, in response to SP stimulation, an upregulation of interleukin 17A (IL-17A) mRNA expression in preadipocytes and an increase in IL-17A receptor (IL-17RA) mRNA have been observed in colonic tissue [53]. This resulted in an overall increase in the release of proinflammatory cytokines and a concomitant decrease in anti-inflammatory cytokine secretion, changing the cytokine balance in favor of inflammation [53]. Additionally, in an experimental colitis model, gastrointestinal neurotensin (NT) synthesis (a neuropeptide closely involved in intestinal inflammation) and its corresponding receptor NTR1 were significantly upregulated in mesenteric fat, leading to IL-6 secretion by preadipocytes [54]. These findings suggest that creeping fat, rather than expressing genes involved in lipid metabolism, displays immunological characteristics that contribute to reciprocal signaling with the mucosal immune system [55]. The excessive expression of chemoattractant molecules plays a crucial role in coordinating the recruitment of immune cells, ultimately leading to the development of tertiary lymphoid organs (TLOs) within adipose tissue, which actively sustain intestinal inflammation [55]. As a result, creeping fat has the potential to worsen intestinal inflammation by exacerbating transmural injury and precipitating fibromuscular proliferation, which are closely related to Crohn’s disease complications [55].

## 5. Surgical and Histological Evidence for the Importance of Creeping Fat

Studies on Crohn’s disease have mainly focused on alterations occurring within the intestinal wall and the accompanying inflammatory processes in the mucosa and submucosa [56,57]. However, recent evidence indicates that mesenteric tissue plays an active role in Crohn’s disease rather than being a passive bystander [36,58]. Notably, an increased area of visceral fat was identified as an independent risk factor for postoperative disease recurrence in patients with Crohn’s disease [59]. This finding was further supported by studies in diverse ethnic populations [60]. The correlation between expanding mesenteric adipose tissue and markers of inflammation, intestinal wall thickening, and transmural inflammation explains, to a certain extent, the association between creeping fat and a complex disease course [61,62,63]. Additionally, the remodeling of lymphatic vessels and bacterial translocation to mesenteric adipocytes and lymph nodes perpetuate inflammation in the mesentery and intestine, aggravate disease activity, and closely correlate with postoperative disease recurrence [64,65,66]. The intricate interactions between stromal cells and immune cells suggest a pivotal role for the mesentery in the course of Crohn’s disease [36,37,67]. Intuitively, mesentery-based surgery could improve disease outcomes by mitigating these pro-inflammatory interactions. Previous assessments of surgical procedures, including hand-sewn or stapled anastomosis and broad vs. restricted bowel resection, have not shown any clear benefits in preventing postoperative recurrence in patients with Crohn’s disease [68,69]. However, studies suggest that extensive mesenteric resection may confer some advantages. In the mesocolic-resection approach, where both the mesentery and intestine are considered, the mesentery transition point serves as the landmark for intestinal division [65]. This approach minimizes the challenges associated with dividing acutely inflamed mesentery, which typically leads to bleeding [65]. Concerns about the potential for hematoma and hemorrhage due to wide mesenteric resection in the presence of considerable inflammation, adhesions, and abscess formation have been reported [70,71]; however, there is evidence supporting that mesentery inclusion during ileocolonic resection is considered safe [67,71]. In particular, Coffey et al. [67] showed that the inclusion of the mesentery as part of the surgical excision of the appropriate affected segment of bowel resulted in a significant reduction in the surgical recurrence rate. Particularly, in a total of 30 patients who underwent standard ileocolic resection (the mesentery was divided flush with the intestine), 40% required reoperation for a Crohn’s-related indication [67]. In contrast, in a total of 34 patients who underwent resection in which mesentery was also resected, only 2.9% (*p* = 0.003) required reoperation [67]. In addition, it has been reported that a mesenteric severity index, which includes histological assessment of fat envelopment and mesenteric thickening, correlates strongly with mucosal and Crohn’s disease activity indices [67]. 

On the other hand, a recent meta-analysis examining the efficacy of Kono-S, a procedure involving an antimesenteric, functional, continuous, hand-sewn anastomosis with preservation of the mesentery, yielded equally impressive results [72]. Notably, the postoperative recurrence rate in patients treated with the Kono-S technique was 0–3.4% vs. 15–24.4% in the standard anastomosis group. Paradoxically, both the mesentery-sparing Kono-S procedure and enterectomy in conjunction with radical mesenteric resection appear to improve long-term outcomes. The fact that both techniques isolate the anastomosis as much as possible from the affected mesenteric tissue may explain this discrepancy to some extent [72]. Table 1 summarizes the data related to postoperative recurrence after surgical resection.

Intraoperatively, surgical assessment of adipose tissue engagement helps identify areas of bowel involvement and define resection margins in Crohn’s disease [34,62]. Macroscopically, a transition zone can be seen in the mesentery where normal tissue is gradually replaced by creeping fat. Substitution of the normal bowel wall by mucosal and mural lesions coincides with the overlying mesenteric transition zone [34,67]. Creeping fat develops in topographic association with inflammatory lesions, forming visually arresting finger-like projections that encircle the affected bowel [67,73]. At the same time, mesenteric connective tissue lesions spread uninterruptedly into the adjacent musculature or even deeper intestinal layers [67]. Moreover, transmural inflammation is the most prominent of the histopathological entities associated with fat encasement [62]. Another study has shown that acute and chronic inflammatory features, including depth of wall damage as well as abundance of lymphoid aggregates, are significantly associated with the degree of serosal creeping fat [22,62].

According to the Montreal classification, the behavior of Crohn’s disease can be divided into three main categories, namely B1: non-restrictive, non-penetrative; B2: restrictive; and B3: penetrative [74]. Interestingly, recent data demonstrate that creeping fat may also influence the phenotypic manifestation of Crohn’s disease [24]. There is increasing evidence that muscle proliferation promotes the formation of strictures [75,76,77]. Chen et al. [75] recognized hypertrophy of muscularis propria as the predominant histological feature of stricture tissue, followed by the proliferation of submucosal smooth muscle cells. A later study reported that fibrostenosis was mainly associated with abnormal expansion and architectural disorder of the muscularis mucosa [76]. Creeping fat and the underlying muscle layers are not only anatomically close, but also functionally interdependent. Specifically, subserosal creeping fat is a major source of long-chain free fatty acids that induce selective smooth muscle cell expansion of the adjacent muscularis propria through a process requiring the carnitine palmitoyltransferase-1 pathway and sphingosine biosynthesis [78]. Conversely, disease-activated muscularis propria cells synthesize an extracellular matrix platform that promotes preadipocyte migration from adjacent mesenteric adipose tissue [79]. The muscle-derived scaffold is composed of numerous proteins, of which fibronectin was found to be under the control of the pro-fibrotic mediator transforming growth factor-β1 (TGF-β1) [79]. Moreover, fibronectin has been identified as a major driver of preadipocyte migration and differentiation via a fibronectin-mediated migration of preadipocytes through the fibronectin/α5β1 integrin signaling pathway, which accelerates the development of creeping fat [79]. In light of this, the adipocyte-smooth muscle cell axis appears to have a significant impact on stricture formation during the course of Crohn’s disease [24].

## 6. Creeping Fat: The Radiological Approach

Adipose tissue is distributed in two main compartments, namely subcutaneous (SAT) and visceral adipose tissue (VAT); the latter includes mesenteric fat. Advances in cross-sectional imaging have made it possible to visualize and quantify adipose tissue depots with excellent accuracy [80]. In particular, magnetic resonance imaging (MRI) and computed tomography (CT) are now considered the reference methods for measuring and differentiating VAT and SAT, as they allow multidimensional visualization of fat distribution [80,81]. Patients with Crohn’s disease tend to have increased intra-abdominal adiposity compared with healthy controls [26,82,83]. Notably, numerous imaging studies report that visceral fat content significantly correlates with disease activity, Ref. [84]; inflammatory biomarkers, [27,85]; endoscopic activity, Ref. [59]; postoperative adverse outcomes, [59,60,86,87]; poor response to treatment, [84]; shorter remission intervals, Ref. [26]; impaired quality of life, Ref. [27]; and complicated disease phenotype [25,26,27]. In contrast, some studies suggest that visceral adiposity does not predict postoperative outcome in Crohn’s disease [88] or even protect against adverse outcomes [89]. The quality of relevant studies, which varies from low to moderate, may explain these conflicting data; most of them are retrospective in design, include inadequate sample sizes, and use heterogeneous (CT or MRI) and non-standardized methods [the assessment level ranges from T10 to L5 even in CT-based studies] to measure VAT [82]. At the same time, the VAT phenotype is not universal, e.g., the fat-wrapping in Crohn’s disease differs significantly from obesity-related VAT, suggesting a non-linear correlation between VAT and Crohn’s disease [90]. Although the extent of VAT can be accurately measured, the efficacy of imaging techniques to assess the amount of creeping fat separately from VAT is controversial. Distinguishing between physiologic perienteric and creeping fat can be difficult because their radiodensity and signal are similar on CT and MR imaging, respectively; however, fat stranding and edema may be helpful in this regard [24,91]. Therefore, the proliferation of adipose tissue as depicted on imaging studies is thought to be relevant to, but not the same as, the histopathologic/surgical definition of fat-wrapping [24].

Nevertheless, the growing interest in fat-wrapping in Crohn’s disease has rejuvenated radiologic research in this area. Table 2 presents data on the assessment of creeping fat and its impact on the course of patients with Crohn’s disease.

Rimola et al. [97] defined creeping fat as an increased volume of adipose tissue surrounding an affected bowel segment using an MR enterography protocol. Their results showed that creeping fat persisted even during endoscopic remission, suggesting that fat-wrapping is indicative of ongoing injury rather than active inflammation [97]. Alternatively, creeping fat may be delineated as an expansion of mesenteric adipose tissue surrounding the affected bowel, leading to defective separation or displacement of adjacent bowel loops [73,98]. A prospective MR enterography-based study, using the latter definition, showed satisfactory interobserver agreement [99]. The presence of creeping fat at baseline was an independent predictor of failure to achieve a cure of severe inflammation after 48 weeks of treatment with tumor necrosis factor-a (TNF-α) inhibitors [99]. In this regard, another MR imaging study reported that the prevalence of creeping fat in a Crohn’s disease cohort was 21.1% [95]. In addition, creeping fat was independently associated with aggressive disease progression leading to debilitating complications, severe bowel damage, and increased risk of surgery [95]. These findings agree with a very recent study that showed a significant association between a qualitative assessment of creeping fat and Crohn’s disease severity [100]. In this study, the Magnetic Resonance Index of Activity (MaRIA) score was used for the evaluation of Crohn’s disease activity [100]. However, mesenteric fat changes and creeping fat were not included in this score, highlighting the fact that more attention should be given to creeping fat assessment in patients with Crohn’s disease [100].

Similarly, the use of CT-based techniques has just begun to revolutionize the field of noninvasive fat-wrapping assessment. The proliferation of creeping fat is accompanied by increased angiogenesis, leading to hypervascularity, which is recognizable as “comb sign” in CT enterography [91]. Therefore, increased vascular density surrounding the intestinal lumen can be used to differentiate from fat-wrapping tissue. In this regard, Li et al. [92] developed and validated a novel CT enterography-based mesenteric creeping fat index (MCFI) to mitigate the confounding effect of normal retroperitoneal and perienteric adipose tissue. Radiologically determined MCFI showed satisfactory interobserver agreement and strong correlation with macroscopic creeping fat and histologic fibrosis scores determined in surgically resected segments [92]. In addition, MCFI was significantly associated with muscularis mucosa and muscularis propria thickness and showed high diagnostic accuracy in assessing the severity of fibrostenosis, surpassing previous indices, namely fibrofatty proliferation score and visceral to subcutaneous fat ratio [92]. These results confirm the hypothesis of the synergy of creeping fat and intestinal muscle in promoting stricture in Crohn’s disease [24]. Another study quantifying creeping fat developed a formula to calculate the slope of the Hounsfield unit curve (λHU) based on the energy spectrum CT [94]. Specifically, the CT attenuation coefficients were plotted at different energy levels (from 40 keV to 140 keV) and the resulting spectral HU curves were used to specifically identify the tissue of the studied region and to distinguish creeping fat from VAT. The authors reported that the λHU of creeping fat strongly correlated with the severity of bowel inflammation, could predict the formation of strictures, and could identify inflammatory lesions with higher accuracy compared with other established indices [94]. In addition, fat-wrapping appeared to occur more frequently around unaffected intestinal segments in patients with Crohn’s disease compared with healthy controls, suggesting that early noncanonical fat proliferation may precede the onset of wall damage [94].

## 7. The Role of Artificial Intelligence Technology

Artificial intelligence (AI), including machine learning and deep learning, has emerged as a breakthrough, simulating human intelligence by machines, thus enabling the processing of data across all sectors [101]. AI allows computer systems to recognize, quantify, and interpret potential relationships among variables using algorithms, which is a critical tool for physicians [102]. The increasing data derived by electronic datasets; the development of multi-omics analyses including genomics, transcriptomics, proteomics, and metagenomics; and the rapid progress in imaging modalities have greatly contributed to the delineation of mechanistic insights involved in inflammatory bowel disease. Studies have shown that the integration of AI technology with endoscopy may improve the diagnostic performance, as AI was proved more accurate and faster in the detection of subtle lesions compared with endoscopists [103]. Moreover, combining AI with capsule endoscopy may provide a useful tool for assessing small-bowel and colonic lesions [104], especially for patients with non-obstructed small-bowel stenosis [105]. The examination of a state-of-the-art deep learning network for the detection of strictures in capsule endoscopy images exhibited a high accuracy of 93.5% in detecting stenosis and an excellent differentiation between strictures and ulcers (AUC = 0.942) [105].

In parallel, AI-based quantitative imaging analysis has been suggested as a reliable and rapid technology for the segmentation of abdominal adipose tissue [106]. Different approaches using various AI algorithms for both CT and MR have been recommended for the segmentation of abdominal fat, allowing rapid results with great accuracy (even better than manual segmentation) [106]. Accurate and less variable abdomen assessment could also be provided by AI as regards the single-slice method for VAT and SAT segmentation [107].

Evaluation of perienteric fat by AI technology or by computer-based radiomics, especially on MR images, may provide critical evidence into the processes taking place in this tissue that are visually unclear. A recent study proposed the use of deep learning-based AI for prediction of postoperative recurrence of Crohn’s disease [108]. Algorithms based on convolutional neural network classified the intestinal histologic images with great accuracy (AOC = 0.995) in accordance with the postoperative recurrence of Crohn’s disease [108]. Moreover, subserosal adipose tissue showed the most accurate prediction detectable by AI. This finding is consistent with studies associating the hypertrophied creeping fat tissue on abdominal CT images with early postoperative recurrence in Crohn’s disease [59].

The use of AI technology in inflammatory bowel disease research could facilitate the extraction of quantitative data from digital images, allowing physicians to obtain important information with minimal cost to healthcare, greatly improving the baseline endoscopic characteristic assessment, the therapeutic interventions, and the clinical outcomes. However, the existence of studies reporting significant heterogeneity in AI approaches highlights the urgent need for unbiased validation studies in order to ensure AI’s incorporation into clinical practice.

## 8. Conclusions—Future Perspectives

Creeping fat seems to critically contribute to Crohn’s disease pathogenesis and underlies disease severity and outcomes. The current understanding of creeping fat is restricted, as this tissue displays a platform of highly intricate interactions between various cell subsets, bacteria, and gut antigens [109]. Creeping fat development occurs in association with inflammatory lesions, forming visually arresting finger-like projections that surround the affected bowel. During Crohn’s disease progression, the response of mesenteric adipose tissue to the release of inflammatory mediators and invasion of bacteria through the intestinal mucosa results into the development of creeping fat. Thus, the complex interplay between the translocating bacteria and the immune cells in adipose tissue warrants further investigation. 

The question of whether mesenteric adipose tissue contributes to the induction of inflammatory-mediated intestinal injury or serves as a defensive barrier remains elusive. Data support that creeping fat may influence the phenotypic manifestation of Crohn’s disease. Emerging evidence based on MCFI displays that the severity of creeping fat is positively related to intestinal fibrosis, thickness of muscularis propria, and intestinal stricture degree, suggesting that MCFI may become a non-invasive tool for the assessment of intestinal and mesentery fibrosis in Crohn’s disease. With the advent of machine learning algorithms and computational radiomics, imaging techniques are undoubtedly becoming indispensable tools to evaluate and determine the complex role of creeping fat in Crohn’s disease. Beyond the validation of imaging techniques’ accuracy to precisely determine the creeping fat, well-designed studies are needed to investigate the intricate role of this tissue in Crohn’s disease. Special focus should be given to the impact of creeping fat on the response to therapeutics, which may help in exploring new treating methods. In parallel, it will be intriguing to investigate the differences in the cellular composition of creeping fat among patients who undergo specific immune-suppressive treatments. A better understanding of the effects of stromal vascular fraction on the function and differentiation of adipocytes in creeping fat is also of critical importance. The delineation of these inquiries is essential for comprehending the impact of creeping fat on the clinical progression of Crohn’s disease and assessing the potential benefits of surgically removing creeping fat to achieve clinical remission.

## Figures and Tables

**Table 1:** Summary of information regarding postoperative recurrence after surgical resection in patients with Crohn’s disease.

Authors (Ref.)	Year	Number of Patients	Type of Study	Outcomes
Coffey et al. [67]	2018	158	Prospective cohort study	- Inclusion of mesentery in surgical excision of bowel → surgical recurrence rate reduction - From 30 patients who underwent standard ileocolic resection → 40% required reoperation - From 34 patients who underwent resection including excision of the mesentery → 2.9% required reoperation
Li et al. [65]	2018	63	Prospective study	- Creeping fat was associated with increased mesenteric lymphatic vessel density in the proximal margin - Disease recurrence was associated with increased mesenteric lymphatic vessel density of the proximal mesenteric margin at the time of resection
Li et al. [71]	2020	116	Multicenter, randomized controlled trial	Ongoing trial examining: - Primary ileocolic resection with extensive mesenteric excision vs. conventional ileocolic resection with limited mesenteric excision
Alshantti et al. [72]	2021	896	Meta-analysis	- Kono-S anastomosis was related to a reduced incidence of endoscopic and surgical recurrence vs. the standard anastomosis group (0–3.4% vs. 15–24.4%, respectively)

**Table 2:** Summary of information regarding creeping fat assessment and its impact on the course of the disease in Crohn’s disease.

Authors (Ref)	Year	Number of Patients	Type of Study	Index	Method Description	Results
Erhayiem et al. [61]	2011	50	Retrospective cohort study	Visceral to subcutaneous fat area ratio	Cross-sectional scan at the level of the umbilicus was used to determine the areas of subcutaneous and visceral fat. MFI was defined as the ratio of areas of visceral to subcutaneous fat.	- Visceral fat area was highly associated with the development of stricturing or fistulizing Crohn’s disease. - High ratio of areas of visceral to subcutaneous fat was associated with aggressive Crohn’s disease.
Li et al. [92]	2021	91	Retrospective cohort study			- No association was observed between visceral and subcutaneous fat area ratios with histological findings. - No association was observed between visceral and subcutaneous fat area ratios with intestinal stricture index.
		30	Prospective cohort study			
Sakurai et al. [93]	2017	41	NR	Fibrofatty proliferation score	Fibrofatty proliferation score was evaluated according to the increase in mesenteric fat degree around the affected intestine and the degree of displacement of the adjacent intestine. A score of 0-1-2 points corresponded to none, mild, and moderate-to-severe, respectively.	- Weak association was observed between fibrofatty proliferation score and muscularis mucosae thickness. - No association was observed between fibrofatty proliferation score and other histological findings. - Weak association was observed between fibrofatty proliferation score and intestinal stricture index.
Coffey et al. [68]	2018	158	Prospective cohort study	Mesenteric disease activity index	Mesenteric disease activity index was developed using fat wrapping and mesenteric thickening. Fat wrapping was graded in accordance with the proportion of intestinal circumference affected. Mesenteric thickening was graded in accordance with vascular and avascular mesenteric regions appearance.	- Mild mesenteric disease matched to minimal fat wrapping. - Moderate mesenteric disease matched to <25% fat wrapping. - Severe mesenteric disease matched to >25% fat wrapping.
Feng et al. [94]	2018	80	Retrospective cohort study	Energy spectral computed tomography	The slope of the λHU was measured and calculated on energy spectral CT images.	- Creeping fat λHU in patients with Crohn’s disease increased along with the intestinal inflammation severity. - Creeping fat λHU around the intestinal segments without lesions in Crohn’s disease was significantly larger compared with controls. - λHU was more accurate for the detection of Crohn’s disease inflammatory lesions compared with calculating visceral fat.
Althoff et al. [95]	2019	90	Retrospective observational cohort study	Small bowel magnetic resonance imaging	Site of inflammation and involvement of mesenteric and peri-intestinal fat were taken into consideration.	- Creeping fat was strongly associated with a complicated Crohn’s disease course, bowel damage, and abdominal surgery occurrence. - Creeping fat was associated with stenoses development, but not fistula.
Li et al. [92]	2021	91	Retrospective cohort study	MCFI	Creeping fat severity was graded based on the extension of mesenteric fat around the intestinal circumference. MCFI accuracy was evaluated by comparing it with the creeping fat degree in surgical specimens.	- Strong association between MCFI and the extent of macroscopic fat wrapping. - Strong association between MCFI and histological degree of fibrostenosis.
		30	Prospective cohort study			
Meng et al. [96]	2022	174	Retrospective multicenter study	Model 1	MCFI	- Model 3 presented the most satisfactory clinical practicability and optimal performance for the differentiation between non-mild and moderate-to-severe fibrotic intestinal strictures in patients with Crohn’s disease compared with Model 1 and Model 2.
				Model 2	Mesenteric oedema and MCFI	
				Model 3	Mesenteric oedema, MCFI, and disease duration	

Abbreviations: Ref, reference; MFI, mesenteric fat index; NR, not reported; λHU, Hounsfield unit curve; CT, computed tomography; MCFI, mesenteric creeping fat index.

## Data Availability

Not applicable.
